# Effect of Short-Term High-CO_2_ Treatments on the Quality of Highbush and Rabbiteye Blueberries During Cold Storage

**DOI:** 10.3390/plants13233398

**Published:** 2024-12-03

**Authors:** Jose David Toledo-Guerrero, Maria Dolores Álvarez, Beatriz Herranz, M. Isabel Escribano, Carmen Merodio, Irene Romero, M. Teresa Sanchez-Ballesta

**Affiliations:** 1 Department of Characterization, Quality and Safety, Institute of Food Science, Technology and Nutrition (ICTAN-CSIC), Jose Antonio Novais 6, 28040 Madrid, Spain; j.toledo@ictan.csic.es (J.D.T.-G.); mayoyes@ictan.csic.es (M.D.Á.); herranzh@vet.ucm.es (B.H.); escribano@ictan.csic.es (M.I.E.); merodio@ictan.csic.es (C.M.); 2 Department of Food Technology, Veterinary Faculty, Complutense University, Avda/Puerta de Hierro, s/n, 28040 Madrid, Spain

**Keywords:** cell wall, gene expression, low temperature, mechanical properties, *Vaccinium*

## Abstract

The global demand for blueberries has increased due to their health benefits, but postharvest losses, particularly firmness loss and decay, present significant challenges. This study evaluated the effects of high CO_2_ concentrations (15% and 20%) applied for 3 d at 1.0 °C on highbush (cv. ‘Duke’) and rabbiteye (cv. ‘Ochlockonee’) blueberries, with a focus on quality maintenance during cold storage. The quality parameters evaluated included titratable acidity, pH, total soluble solids, weight loss, and decay. The effect of gaseous treatments on firmness was analyzed using mechanical parameters and the expression of genes related to cell wall integrity (*XTH23*, *PL8*, *PG*, *PM3*, *EXP4*, and *VcGH5*). Treatment efficacy varied between species. High CO_2_ levels reduced decay in both cultivars, but only the highbush cultivar (‘Duke’) showed improvements in firmness. In ‘Duke’, CO_2_ treatments affected the expression of *XTH23*, *PL8*, and *GH5*, while the role of *PG* and *PME* in maintaining firmness was minimal, with no significant differences between treatments. In ‘Ochlockonee’, CO_2_ effectively reduced weight loss but did not improve firmness. In conclusion, these results highlight the need for tailored postharvest strategies for different blueberry cultivars and suggest that short-term high CO_2_ treatments may effectively prolong the postharvest life of highbush blueberries.

## 1. Introduction

Blueberry (*Vaccinium* spp.) is a soft fruit popular with consumers for its distinctive taste and high nutrient content, including phenolic acids and anthocyanins [1,2]. However, once harvested, the quality of blueberries is compromised due to their high respiration rate, softening, and high susceptibility to fungal attack [3]. Although it is recommended that blueberries be stored at a temperature close to 0 °C to prolong their postharvest life, this condition alone is not sufficient to maintain quality as it can also promote softening, pedicel pitting, pericarp and pulp adhesion, and fungal decay [4]. While several studies have explored the development of technologies combined with low temperatures to preserve the quality of soft berries after harvest, much of the focus has been on strawberries [5,6,7,8]. High CO_2_ concentrations in the range of 15 to 20% have been shown to mitigate fungal attack and reduce respiration rates and softening in blueberries, thereby extending their shelf life [3]. Various postharvest preservation methods such as controlled atmosphere (CA), modified atmosphere packaging (MAP), heat shock, sulfur dioxide, ethanol, and edible coatings have been used to extend the shelf life of blueberries [9,10,11,12,13,14,15]. Previous research has shown that short-term exposure (2–3 d) to 20% CO_2_ is effective in maintaining the quality of table grapes and strawberries stored at low temperatures [6,16]. Although these short-term gaseous treatments may reduce costs compared to traditional CA by minimizing the duration of exposure to non-atmospheric conditions, their effectiveness in blueberries has not been studied.

When assessing the efficacy of postharvest treatments on blueberries, firmness is one of the most important parameters to consider. On the other hand, firmness is a genetically controlled trait [17], so the genotype of a given blueberry is a factor influencing it. Therefore, changes in postharvest firmness can be very different between the most widely cultivated blueberry species in the world, the highbush (*V. corymbosum* L.) and the rabbiteye (*V. ashei* Reade) blueberries. Rivera et al. [18] found that mechanical parameters from penetration tests effectively assess the firmness of blueberries, revealing how postharvest-controlled atmosphere conditions and genetic variation affect quality. The review highlighted that this method is valuable for distinguishing between berries, influenced by factors such as genetic differences [19], postharvest conditions [20,21], and maturity stage [22]. In previous research, Rivera et al. [22] investigated key mechanical properties related to moisture loss in two blueberry cultivars, ‘Nui’ and ‘Rahi’, using double compression determined by the texture profile analysis (TPA) and puncture tests. Their results showed a strong correlation between water loss and hardness measured by the TPA test, as well as the displacement at the berry skin break from the puncture test. Consequently, these mechanical parameters can serve as effective tools for quantifying changes in stored blueberries and tracking ripening progress in different cultivars. More recently, Sanchez-Ballesta et al. [23] used different mechanical parameters from a penetration test to measure the quality loss of 10 different commercial blueberries due to softening upon arrival at the retail market, just before consumer purchase, in terms of firmness. They found significant differences in the values of mechanical parameters associated with the skin-breaking (maximum skin-breaking force, distance required to break the skin, slope of the curve corresponding to skin penetration to break, and work required to break the berry skin).

The decrease in firmness is primarily caused by water loss and changes in the structure and composition of the cell wall [24]. During postharvest, fruit softening is closely linked to the disassembly of pectin and cellulose, which occurs gradually during storage. The degradation of cell wall components is carried out by the concerted action of several cell wall pectin-degrading enzymes, such as polygalacturonase (PG), cellulase (CL), β-galactosidase (β-Gal), and pectin methyl esterase (PME) [25,26,27]. Transgenic silencing of some of these genes reduced softening and increased the postharvest life of strawberries and apples [28,29]. Recently, *FaPG1* knockout strawberry plants generated using the CRISPR/Cas9 system showed a reduced postharvest softening rate and fungal decay [30], suggesting that this gene plays a key role in maintaining firmness during postharvest.

In the case of blueberries, several studies have shown that the application of postharvest treatments helps to maintain fruit firmness and modulate the expression of genes encoding cell wall-modifying enzymes, with *PG* being one of the most studied. For example, the postharvest application of putrescine delayed fruit softening and inhibited *VcPG1* expression levels [31]. Similarly, ethanol vapor delayed the softening of blueberries and downregulated *PG* gene expression [32]. However, the effect of the application of short-term gaseous treatments with high levels of CO_2_ on blueberry quality, particularly firmness, and the modulation of cell wall-modifying gene expression, remains unknown. To investigate the role of short-term CO_2_ treatments in maintaining quality and controlling decay and water loss in blueberries, this study applied two gaseous treatments with different concentrations of CO_2_ (15% and 20%) for 3 d at 1.0 °C to highbush (cv. ‘Duke’) and rabbiteye (cv. ‘Ochlockonee’) blueberries. The effects of these gaseous treatments on the mechanical properties and the expression of genes related to cell wall-modifying enzymes, such as *xyloglucan endotransglucosylase/hydrolase* (*XTH23*), *pectate lyase* (*PL8*), *PG*, *PME3*, *expansin* (*EXP4*), and *glycosyl hydrolase* (*GH5*), were also evaluated during low-temperature storage for up to 29 d.

## 2. Results

### 2.1. Effect of Short-Term High-CO_2_ Treatments and Storage at 1.0 °C on Blueberry Quality and Weight Losses

**Figure 1:**
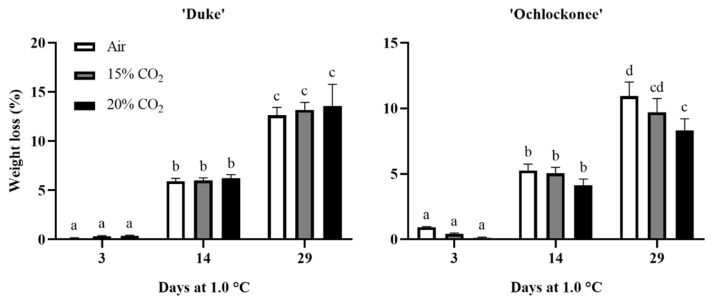
Weight loss of non-treated, 15%, and 20% CO_2_-treated ‘Duke’ and ‘Ochlockonee’ blueberries stored at 1.0 °C for 29 d. Values are the means of three replicate samples ± standard error. Different letters within each column indicate that the means are statistically different according to the Tukey-b test (*p* < 0.05).

The effect of short-term gaseous treatment on the quality parameters analyzed depended on the cultivar (‘Duke’ and ‘Ochlockonee’) and the percentage of CO_2_ used. For ‘Duke’ fruit, weight loss during cold storage increased similarly for both treated and non-treated blueberries. However, although low temperatures increased weight loss in ‘Ochlockonee’ fruit, the application of 20% of CO_2_ significantly reduced it by the end of cold storage compared to non-treated fruit (Figure 1).

The soluble solids content (SSC) of non-treated ‘Duke’ fruit and those treated with 15% or 20% CO_2_ decreased significantly after 3 d at 1.0 °C (Table 1), with similar levels in all three cases. However, while the SSC content of fruit treated with 15% and 20% CO_2_ remained unchanged from day 3 to the end of storage, it increased in non-treated fruit to levels similar to those observed in freshly harvested fruit.

Titratable acidity (TA) increased in both non-treated and CO_2_-treated ‘Duke’ fruit on day 3. However, significant differences were observed between treated and non-treated ‘Duke’ fruit at the end of the storage. In non-treated fruit, a decrease in TA was observed on day 29 compared to day 0 (Table 1). In treated fruit, TA varied according to the percentage of CO_2_. ‘Duke’ blueberries treated with 15% CO_2_ showed an increase in TA on day 29 compared to freshly harvested fruit, whereas blueberries treated with 20% CO_2_ reached values similar to those observed on day 0. The maturation index (MI), which is the ratio of SSC/TA, was affected by these changes, resulting in a significant decrease after 3 d in all conditions analyzed. Non-treated ‘Duke’ fruit showed a significant increase in MI compared to freshly harvested fruit, while fruit treated with 15% CO_2_ showed a decrease, and there was no change in fruit treated with 20% CO_2_. Regarding pH, this parameter increased significantly during the storage of ‘Duke’ fruit at 1.0 °C under all conditions analyzed, except for fruit treated with 15% CO_2_ after 3 d.

For ‘Ochlockonee’ fruit, it should be noted that the SSC content of non-treated and 15% CO_2_-treated fruit increased significantly by the end of the storage compared to freshly harvested fruit (Table 2). However, no changes were observed in fruit treated with 20% CO_2_. In addition, the TA of non-treated and 20% CO_2_-treated fruit increased during the storage at low temperatures, whereas it decreased in fruit treated with 15% CO_2_. These changes in SSC and TA led to a decrease in MI in both non- and 20% CO_2_-treated fruit, whereas it increased in fruit treated with 15% CO_2_. In addition, the pH increased significantly in non-treated and 15% CO_2_-treated ‘Ochlockonee’ fruit stored at 1.0 °C for up to 29 d compared to freshly harvested fruit, similar to ‘Duke’ fruit, and remained unchanged in 20% CO_2_-treated fruit.

The application of 15% and 20% of CO_2_ at low temperatures controlled the increase in total decay observed in non-treated fruit of both cultivars (Table 1 and Table 2). However, the short-term 20% CO_2_ treatment was more effective in reducing total decay in both cases.

### 2.2. Mechanical Parameters

**Figure 2:**
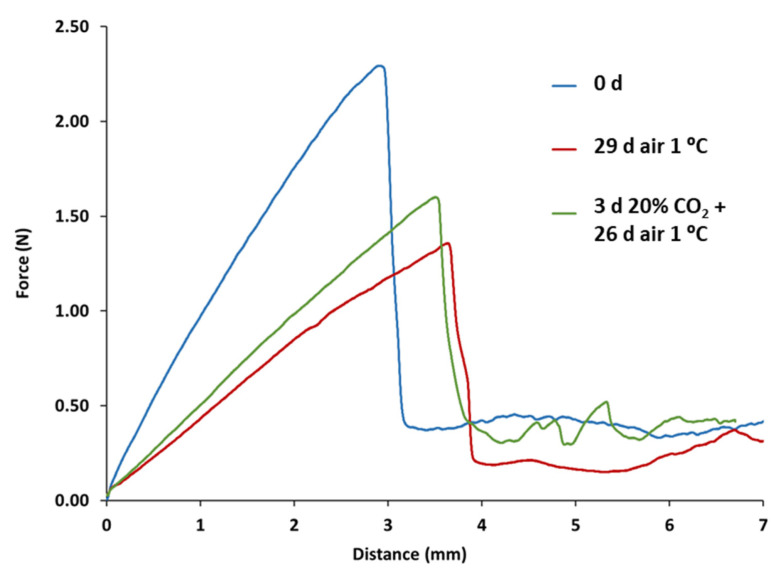
Examples of penetration force–distance curves derived from the penetration test for ‘Duke’ blueberries at the time of harvest and for non-treated and 20% CO_2_-treated fruit after 29 d of storage at low temperature.

The study revealed significant differences in the shape of the force–distance curves (Figure 2) and the mechanical properties between CO_2_-treated and non-treated blueberries, as well as in the values of the various mechanical properties derived from them (Table 3 and Table 4). For example, the penetration force–distance curves obtained for ‘Duke’ blueberries at harvest and for non-treated and 20% CO_2_-treated fruit after 29 d of storage at 1.0 °C are shown in Figure 2. The profiles show a noticeable peak force corresponding to the breaking of the skin or epidermis, followed by a large drop in force and then a continuous flow with a much lower force than the maximum force when the probe penetrates the flesh. This suggests that the strength of the skin has a greater effect on the texture of blueberries than the flesh. However, it should be noted that after 29 d of storage at low temperature, the slope and the distance or displacement at skin break show a decrease and an increase, respectively, compared to the values of the berries determined after the 20% CO_2_ treatment (Table 3). In addition, the maximum skin-breaking force, as well as the slope at skin breaking and the penetration work of ‘Duke’ blueberries, were also significantly lower in non-treated blueberries than in those treated with 15% and 20% CO_2_ for 29 d (Table 3).

After 3 and 14 d of storage, the skin-breaking force of ‘Duke’ blueberries was lower for berries treated with both CO_2_ levels than for fruit stored in the air (Table 3). However, after 29 d of storage at a low temperature, berries stored in the air were significantly softer than those treated with both CO_2_ levels. In contrast, for ‘Ochlockonee’ blueberries, there was no significant difference in skin firmness between the berries at harvest and all the berries stored under the three different storage conditions and times (Table 4). Regarding the slope at skin breaking of ‘Duke’ blueberries (Table 3), only the value corresponding to non-treated berries after 3 d of storage was significantly higher than that of freshly harvested berries and those stored for 3 d at both CO_2_ levels. As observed for breaking force, the lowest slope corresponded to non-treated blueberries for 29 d at 1.0 °C. For ‘Ochlockonee’ fruit (Table 4), the slope at skin breaking on day 3 of storage at 1.0 °C was significantly higher for non-treated and 20% CO_2_-treated fruit than for freshly harvested blueberries. However, at the end of storage, no differences in this parameter were observed between fruit treated with both CO_2_ concentrations and untreated fruit.

Skin-breaking distance values were the only parameter whose values increased with storage time in both treated and non-treated fruit of both cultivars compared to freshly harvested fruit (Table 3 and Table 4). However, while the increase occurred on day 29 in ‘Duke’, an increase was already observed on day 14 for ‘Ochlockonee’. It is also important to note the differences observed between the treated and non-treated samples analyzed. In ‘Duke’, the highest increase was observed in the non-treated fruit stored for 29 d (Table 3). However, while the increase observed in the ‘Ochlockonee’ fruit at 14 d did not show any significant differences between the different samples analyzed, the fruit treated with 20% CO_2_ showed the lowest increase at 29 d (Table 4).

However, for both cultivars, there were very few significant differences in skin breaking observed both at harvest and during storage under the different conditions and time periods analyzed (Table 3 and Table 4). Only the non-treated ‘Duke’ blueberries showed a lower penetration work value at 29 d than at harvest (Table 3).

Finally, a positive and significant correlation was found between force, slope, and work, while a negative and significant correlation was observed between slope and distance in both cultivars. 

### 2.3. Relative Expression of Genes Encoding Cell Wall-Modifying Enzymes

**Figure 3:**
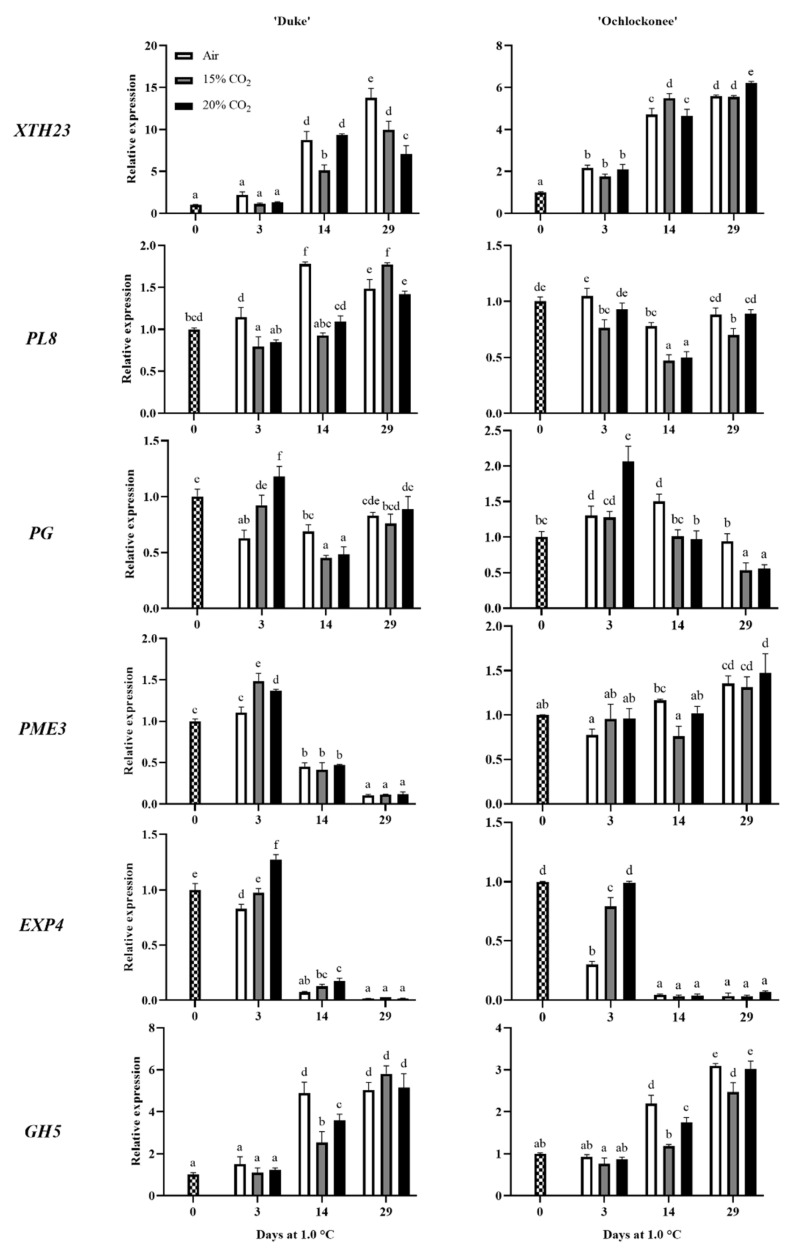
Effect of low-temperature 3-day treatment with 15% or 20% of CO_2_ on the expression of cell wall-related genes in ‘Duke’ and ‘Ochlockonee’ blueberries stored at 1.0 °C for 29 d. Transcript levels were assessed by RT-qPCR and normalized using *EF1* as a reference gene. Results were calculated relative to a calibrator sample (time 0) using the 2^−ΔΔCt^ method. Values are the mean ± SD, *n* = 6. Different letters on bars indicate significant differences using the Tukey-b test (*p* < 0.05).

The temporal expression patterns of six genes encoding cell wall-modifying enzymes were analyzed during low-temperature storage of non-treated and CO_2_-treated ‘Duke’ and ‘Ochlockonee’ blueberries using real-time PCR (Figure 3). Differences in gene expression were observed between ‘Duke’ and ‘Ochlockonee’ fruit.

An induction of *XTH23* expression was observed after 14 d of storage of ‘Duke’ fruit at 1.0 °C, which was higher in both non-treated and 20% CO_2_-treated fruit. However, by day 29, expression decreased in the 20% CO_2_-treated fruit, while it continued to increase in the 15% treated and non-treated fruit, with the latter showing the highest accumulation. In ‘Ochlockonee’ blueberries, expression increased in all the fruit analyzed from day 3, with higher levels in 15% CO_2_-treated fruit at day 14 and in 20% CO_2_-treated fruit at day 29.

Regarding *PL8* expression, it increased only in non-treated ‘Duke’ fruit up to day 14, whereas in CO_2_-treated fruit, expression increased only at the end of storage. The expression pattern was different in ‘Ochlockonee’ fruit, where a decrease in expression was observed in fruit treated with 15% CO_2_ compared to freshly harvested blueberries. However, there was no change in non-treated and 20% CO_2_-treated fruit, except on day 14 when a decrease was also observed.

In the case of *PG*, the storage of non-treated ‘Duke’ fruit at low temperatures reduced its expression compared to freshly harvested blueberries. The application of high levels of CO_2_ resulted in a different pattern of expression at the end of the treatment, depending on the percentage applied. While 15% CO_2_ did not affect *PG* expression, the application of 20% increased the levels. However, in both cases, when the fruit was transferred to atmospheric conditions, a reduction or no change was observed compared to non-treated fruit. On the other hand, the storage of non-treated ‘Ochlockonee’ blueberries at low temperatures temporarily increased *PG* accumulation. In this cultivar, the application of 15% CO_2_ did not affect *PG* expression until day 29, when a decrease was observed. However, short-term treatment with 20% CO_2_ significantly increased *PG* expression at day 3, similar to the response of ‘Duke’ fruit, with levels higher than those of non-treated fruit. Despite this increase, exposure of the 20% CO_2_-treated ‘Ochlockonee’ fruit to atmospheric conditions reduced *PG* accumulation, similarly to the 15% CO_2_-treated fruit.

The expression of *PME3* showed an increase in CO_2_-treated ‘Duke’ blueberries at the end (3 d) of both treatments, while no changes were observed on this day in non-treated fruit compared to freshly harvested fruit. However, from day 14 to the end of storage, the accumulation decreased sharply in all samples. In contrast, no changes in *PME* accumulation were observed in treated and non-treated ‘Ochlockonee’ blueberries until day 29, when expression increased similarly in all samples.

Both cultivars showed a very similar expression pattern for *EXP4.* Notably, after 3 d, the expression was higher in fruit treated with 20% CO_2_ compared to the other samples, either because it increased in ‘Duke’ or remained the same in ‘Ochlockonee’. A drastic decrease in *EXP4* expression was also observed in all samples from day 14 to the end of storage.

Finally, *GH5* also showed a similar expression in both cultivars. On day 3, no changes were observed in all samples analyzed for both cultivars. However, by day 14, a strong increase in gene expression was observed in non-treated samples of both cultivars. Although expression also increased on this day in CO_2_-treated fruit, it was significantly lower. In addition, *GH5* expression continued to increase on day 29, although in this case, it was very similar in all samples analyzed of both cultivars.

## 3. Discussion

Blueberry fruit undergoes a number of physiological and biochemical changes during postharvest storage at low temperatures that result in a loss of quality, primarily due to decreased firmness and fungal decay. Short-term treatments with 20% CO_2_ have been shown to be effective in maintaining quality attributes and reducing total decay in both table grapes and strawberries and in maintaining firmness in strawberries [6,16,33]. However, the effect of these treatments on the postharvest quality preservation of blueberries remains unknown. Therefore, in the present study, as a first approximation, two concentrations of CO_2_ (15% and 20%) were tested and applied for 3 d at 1.0 °C to assess their effect on the quality of blueberries stored for up to 29 d at a low temperature.

Soluble solids, titratable acidity, and pH are indicative parameters of fruit quality and significantly influence sensory characteristics. Differences in these parameters can be observed between cultivars of the two species at harvest, with ‘Duke’ blueberries showing lower SSC and higher TA than ‘Ochlockonee’ (Table 1 and Table 2). Similar results have been reported in previous studies; for example, Rodríguez Madrera et al. [34] indicated that ‘Duke’ fruit had an SSC of 9 and a TA of 0.4%, while ‘Ochlockonee’ fruit had an SSC of 13.1 and a TA of 0.2%. Our results showed changes in SSC, TA, and pH that varied according to cultivar, gaseous treatment applied, and duration of storage at 1.0 °C. Low-temperature storage is known to affect these quality attributes, generally increasing SSC and pH [13,35,36]. However, no consistent pattern has been reported for TA during low-temperature storage, with some authors reporting an increase [37], a decrease [38], or no change [36]. Our results are consistent with these previous findings, showing that both SSC and pH increased in both cultivars at the end of storage at 1.0 °C, while TA decreased in the ‘Duke’ fruit and increased in the Ocklochonee fruit. The application of high levels of CO_2_ influenced these changes, with differences depending on the concentration applied. In particular, the short-term treatment with 20% of CO_2_ generally maintained levels more similar to those recorded in freshly harvested fruit. Furthermore, as expected, a negative correlation was found between TA and pH values, regardless of the storage conditions, although it was significant only in ‘Duke’ fruit (r = −0.710; *p* < 0.01). Regarding pH, Saftner et al. [39] investigated the instrumental and sensory quality attributes of blueberry fruit from ten highbush cultivars, including ‘Duke’, and found significant positive correlations between pH values and both flavor intensity and the overall eating quality. They noted that TA did not correlate with tartness scores or other flavor-related quality measures. Although our study did not use a panel of trained tasters, trials conducted by ICTAN staff showed that the application of high levels of CO_2_ did not result in any unusual odors or flavors. This may be because the pH of CO_2_-treated blueberries is closer to that of freshly harvested fruit, in contrast to the pH increase observed in non-treated blueberries of both cultivars.

When examining the changes in MI, defined as the SSC/TA ratio, a common measure of fruit sweetness and acceptability, it was observed that after 29 d at 1.0 °C, ‘Duke’ fruit treated with 20% of CO_2_ had an MI similar to that of freshly harvested fruit. However, on the same sampling day, storage in the air significantly increased the SSC/TA ratio, whereas the application of 15% CO_2_ decreased it. For ‘Ochlockonee’ fruit stored for 29 d at 1.0 °C, short-term treatment with 15% of CO_2_ resulted in a significant increase in MI compared to freshly harvested fruit, whereas the application of 20% CO_2_ or storage in atmospheric conditions decreased it. Changes in the SSC/TA ratio during storage at low temperatures or CA have been shown to be cultivar-dependent. Forney et al. [13] analyzed the postharvest characteristics of five highbush blueberry cultivars and found that after six weeks of storage in the air at 1.0 °C, the MI decreased in the ‘Jersey’ and ‘Duke’ cultivars but increased in ‘Brigitta’ and ‘Liberty’ or remained unchanged in ‘Aurora’. However, after storage in different CA conditions (10 kPa O_2_ + 10, 12.5, 15, 17.5, and 20 kPa CO_2_), the SSC/TA ratio decreased in the fruit of all cultivars stored in all atmospheres, except in ‘Brigitta’ and ‘Liberty’ stored in 15.0 kPa CO_2_, where it increased. Conversely, this ratio decreased in ‘Lateblue’ blueberries stored in the air or CA (3 kPa O_2_ + 11 kPa CO_2_) [37].

Short-term gaseous treatments were effective in controlling the total decay in cultivars of both species. Similar results were obtained for blueberries stored in CA conditions. Forney et al. [13] showed that CA storage of five highbush blueberry cultivars in atmospheres of 10 kPa O_2_ and 10–20 kPa CO_2_ inhibited fruit decay. However, CA storage of ‘Lateblue’ blueberries at 3 kPa O_2_ and 11 kPa CO_2_ for 60 d was not effective in controlling decay compared to non-treated fruit [37]. Our results showed differences between the concentrations applied, with the 20% CO_2_ treatment being the most effective in controlling decay in both cultivars.

Weight loss in blueberry fruit is an important quality factor and is mainly due to transpiration [40]. In this study, weight loss during cold storage was dependent on the blueberry cultivar, storage time, and the short-term gaseous treatment applied. The application of high levels of CO_2_ was not effective in controlling weight loss in ‘Duke’ fruit, which reached similar levels in all samples by day 29. However, in ‘Ochlockonee’ fruit, the application of the short-term treatment of 20% CO_2_ reduced weight loss after 29 d at 1.0 °C compared to fruit treated with 15% CO_2_ and non-treated ones. Previous studies have shown that short-term treatments of 3 d with 20% CO_2_ at low temperatures were effective in controlling water loss in strawberries and table grapes [6,41,42]. In blueberries, CA storage at high CO_2_ levels was shown to reduce weight loss [13,43]. ‘Duke’ blueberries showed the highest percentage of weight loss, as reported in several studies analyzing different cultivars [13,44], which may be related to the observed differences in the efficacy of the gaseous treatments.

Previous research has shown that weight loss is the main cause of firmness changes in blueberries during postharvest storage [45]. Firmness is a critical quality parameter for fresh blueberries and has a significant impact on consumer perception of quality and postharvest shelf life [18]. The investigation of mechanical properties revealed notable distinctions between CO_2_-treated and non-treated blueberries across both cultivars. The application of both short-term gaseous treatments resulted in a higher maximum skin-breaking force at the end of storage for ‘Duke’ fruit. Although this force was lower than that of freshly harvested fruit, it was significantly higher than that for non-treated blueberries. In addition, the slope at skin breaking in treated blueberries was greater than in non-treated blueberries, indicating less softening of the berries during postharvest storage, thereby confirming previous findings [46]. Moreover, at the end of the storage period, the skin-breaking distance values of the non-treated blueberries were greater than those of the treated blueberries. This observation suggests a lower loss of skin elasticity in CO_2_-treated blueberries at the end of the storage period [18]. It has been reported that the distance at which the berry skin breaks, as determined by the penetration test, increases with greater water loss during storage [46]. Conversely, Chiabrando et al. [47] reported a decrease in total work calculated from the TPA test during cold storage, which is consistent with our results in non-treated fruit. In contrast, the application of 15 and 20% CO_2_ maintained the work to skin break even after 29 d of fruit storage at 1.0 °C, similar to freshly harvested fruit. However, the firmness parameters of rabbiteye blueberries showed less significant differences between treated and non-treated fruit at any sampling point. In this sense, the values for the maximum skin-breaking force and skin-breaking work showed no differences between all the samples analyzed and the freshly harvested fruit. This suggests that short-term gaseous treatments do not significantly affect firmness in the ‘Ochlockonee’ cultivar. This could be related to the fact that firmness is a genetically controlled trait, making the genotype of each blueberry a factor influencing firmness [17].

Fruit softening is associated with changes in the composition and breakdown of the cell wall, mainly due to the disassembly of polysaccharides, which is driven by cell wall-modifying enzymes [48]. Previous studies have elucidated the key role of specific enzymes such as PG, PME, and PL in cell wall degradation in blueberries [49,50,51,52]. In this study, we investigated the relative expression of six genes (*XTH23*, *PL8*, *PG*, *PME3*, *EXP4*, and *GH5*) encoding enzymes involved in cell wall modification during the postharvest storage of blueberries. The levels of these transcripts varied depending on the cultivar and the short-term gaseous treatments applied. *XTH* is known to play a key role in cell wall modification by cleaving and rejoining xyloglucan, which is critical for softening in various fruits [53,54]. For instance, overexpression of *FvXTH9* and *FvXTH6* in strawberries resulted in significantly softer fruit, requiring less force to penetrate the tissue compared to control fruit [55]. Similarly, the overexpression of *XTH8* from persimmon in tomato fruit resulted in reduced firmness [56]. Our results showed that low-temperature storage of ‘Duke’ blueberries increased the expression of *XTH23*. In contrast, the application of high levels of CO_2_, which resulted in firmer fruit compared to non-treated ones, reduced the accumulation of the transcripts compared to non-treated fruit. However, in both treated and non-treated ‘Ochlockonee’ fruit, where exposure to low temperatures reduced firmness, *XTH23* expression increased in all samples, with no major differences observed between them. Previous research has shown that the exposure of strawberries to 18% CO_2_ for 2 d resulted in firmer fruit, together with a downregulation of *XTH30* and *XTH15* expression compared to non-treated fruit. This suggests that the lower expression of *XTH* in CO_2_-treated fruit may be related to the maintenance of key xyloglucan functions in cell wall remodeling, which helps to maintain turgor pressure [57].

Several studies have shown that the inhibition of pectate lyase genes in strawberries and tomatoes has resulted in an increase in fruit firmness [58,59]. In this context, our results showed that the expression of *PL8* in CO_2_-treated ‘Duke’ fruit was significantly repressed by day 14, reaching levels comparable to those found in freshly harvested fruit. In contrast, *PL8* expression increased in non-treated ‘Duke’ fruit, which exhibited lower firmness. Notably, the expression pattern of this gene differed in the ‘Ochlockonee’ cultivar, where both treated and non-treated fruit showed more similar values for the mechanical properties analyzed.

Fruit softening involves the solubilization and depolymerization of pectin, which is driven by changes in the activities of PG and PME [60]. PME demethylates pectin, converting methyl esters into free carboxylic acids, increasing substrate availability for PG, and facilitating fruit softening. Recently, *FaPG1* knockout strawberry plants generated using the CRISPR/Cas9 system showed a reduced rate of fruit softening and increased susceptibility to *Botrytis cinerea* and water loss [30]. Furthermore, Wang et al. [61] showed that exogenous ethylene stimulated blueberry softening and induced the expression of *VcPG*. However, our results indicate that the expression of both *PG* and *PME* does not correlate with the loss of firmness observed in non-treated ‘Duke’ fruit stored at low temperatures. This observation is supported by the significant induction of *PME* expression in fruit treated with 15 and 20% CO_2_ compared to freshly harvested and non-treated fruit. A similar pattern was observed for *PG* expression in fruit treated with 20% CO_2_. A different firmness pattern was observed in ‘Ochlockonee’ fruit compared to ‘Duke’. Transient induction of *PG* was also observed at the end of the 20% CO_2_ treatment, while non-treated fruit displayed a higher *PG* expression from day 14. However, the mechanical properties evaluated did not show significant differences between treated and non-treated fruit.

Research indicates that expansins are associated with tolerance to environmental stresses such as drought, salt, or heat in various plant species [62,63]. For instance, Han et al. [64] reported that overexpression of the wheat expansin gene *TaEXPB23* enhanced oxidative stress tolerance in transgenic tobacco plants, which correlated with increased activities of cell wall-bound peroxidases. In our study, *EXP4* showed a similar expression pattern in both blueberry cultivars, with higher expression levels after 3 d in fruit treated with 20% CO_2_, followed by those treated with 15% CO_2_. In contrast, the non-treated fruit showed a decrease in *EXP4* expression. This trend may indicate that the CO_2_-treated fruit has a greater tolerance to oxidative stress and the postharvest deterioration typically observed in fruit. Similar results were observed in strawberries treated with high levels of CO_2_ for two days followed by storage at low temperatures for up to 7 d, where firmer fruit exhibited higher *EXP4* expression compared to non-treated fruit [57].

GHs are enzymes that modify plant cell wall polysaccharides and are regulated by both developmental stage and environmental stress. For example, the storage of tomatoes at low temperatures has been shown to delay ripening and senescence while preventing the induction of *glycoside hydrolase* expression observed in fruit stored at room temperature [65]. Similarly, long-term cold storage of peaches has been associated with increased expression of *GH5* [66]. In our study, both blueberry cultivars exhibited a similar pattern of *GH5* expression, with induction observed at day 14 in non-treated blueberries stored at low temperatures. In contrast, this expression was controlled in the treated fruit, regardless of the percentage of CO_2_ applied. As with *EXP4* expression, the changes in *GH5* appear to be more closely related to the response of the fruit to low temperatures than to the mechanical properties analyzed.

Analysis of the variables using categorical principal component analysis (CATPCA) revealed differences between the two cultivars (Supplementary Figures S1 and S2). In ‘Duke’ blueberries, two distinct groups were identified based on storage time. One group included samples from time 0 and 3 d, while the other group comprised samples from medium- to long-term cold storage (14–29 d). Notably, the non-treated fruit stored for 29 d did not cluster with the others (Supplementary Figure S1), with this being the sample with the highest values for the MI, displacement at skin break, total decay, and *XTH23* gene expression. Thus, the positive effect of the 3-day gaseous treatment maintaining fruit quality was evident, as these samples, treated and stored at low temperatures for 29 d, clustered with both treated and non-treated fruit from day 14. For the ‘Ochlockonee’ cultivar, the CATPCA results showed groupings based on storage time, with the samples treated with 15% of CO_2_ and stored for 29 d at 1.0 °C being the only ones that were not grouped (Supplementary Figure S1). These samples also exhibited the highest values for MI and displacement at skin breakage. A significant positive correlation was also observed between changes in *XTH23* expression and displacement at skin breakage values, mirroring the findings in the ‘Duke’ cultivar.

## 4. Materials and Methods

### 4.1. Plant Material and Postharvest Treatments

Organic highbush blueberries (*Vaccinium corymbosum* L. cv. ‘Duke’) and rabbiteye blueberries (*Vaccinium ashei* Reade cv. ‘Ochlockonee’) were harvested in June and September 2022, respectively, in Salas, Asturias, Spain (latitude: 43°24′35′′ N, longitude: 6°15′38′′ W, altitude: 243 m). ‘Duke’ and ‘Ochlockonee’ blueberries were harvested at commercial maturity according to grower standards, with SCC at or above 10% and TA between 0.3 and 1.3%. Uniform, disease-free blueberries of both varieties were hand-picked into 125-g solid board eco-friendly packaging punnets (Smurfit Kappa Polska, Warszawa, Poland) including a lid with a NatureFlex™ (Tecumseh, KS, USA) cellulose transparent film window (Futamura Chemical Co., Ltd., Nagoya, Japan). The dimensions of the punnets were 145 mm × 85 mm × 50 mm, and the weight was 16 g. Immediately after harvesting, the fruit was transported to ICTAN (4 h at 20 °C), and on the same day, the punches were randomly divided into 3 batches of 21 punches each and stored in 1 m^3^ methacrylate cabinets at 1.0 ± 0.5 °C and 95% relative humidity. Initially, 3 random punnets were separated, which constituted the group of “time 0”. On the same day as harvesting, one batch was stored under normal atmospheric conditions (non-treated fruit) for a total of 29 d. The second and third batches were stored with a gas mixture containing 15 kPa CO_2_ + 20 kPa O_2_ + 60 kPa N_2_ (15% CO_2_-treated fruit) or 20 kPa CO_2_ + 20 kPa O_2_ + 60 kPa N_2_ (20% CO_2_-treated fruit), respectively, for 3 d at 1.0 °C and then transferred to the air under the same conditions as the non-treated bunches for 26 d. Fruits from three punnets were sampled at different time points (0, 3, 14, and 29 d), frozen in liquid nitrogen, ground to a fine powder, and stored at −80 °C until further analysis.

### 4.2. Quality Assessments

Blueberry juice was extracted using a blender and analyzed for SSC, TA, and pH. SSC was determined using a digital and temperature-compensated refractometer Atago PR-101 (Atago, Co., Ltd., Tokyo, Japan). TA was determined by titration with 0.1 N NaOH to pH 8.2 using the 862 Compact Titrosampler (Metrohm, Oviedo, Spain) and the results were expressed as % citric acid. The pH of the juice was measured on a pH meter with a glass electrode (micropH 2000, Crison, Alella, Spain).

Weight loss was determined by calculating the percentage of the fresh weight difference between the initial and final weights of the punnets on each sampling day, subtracting the weight of the punnet (16 g). The weight of the decayed blueberries was calculated by subtracting the weight of the healthy fruit from the total weight in each biological replicate. Total decay was expressed as the percentage of the decayed fruit with respect to the original weight.

### 4.3. Mechanical Properties

The mechanical properties of blueberries were assessed using a TA.HDPlus texturometer (Stable Micro Systems Ltd., Godalming, UK) fitted with a 5 kg load cell and operated via Texture Exponent Software (version 6.1.20.0). Each test involved placing a blueberry on a flat metal plate with its stem–root axis aligned parallel to the surface. A flat cylindrical stainless-steel probe (2 mm diameter, P/2) was used to penetrate the fruit at its equator to a depth of 50% of the equatorial diameter. The test speed was maintained at 1 mm/s, with a trigger force set at 0.020 N (2 g). Data were collected at a rate of 500 points per second. From the resulting force–distance curves, measurements were taken for maximum skin-breaking force (N), the slope at the breaking point (N/mm), the displacement needed to break the skin (mm), and the work required for the skin to rupture (mJ), following the methodology described by Sanchez-Ballesta et al. [23]. Mechanical parameter values represent the average of 15 berries for ‘Duke’ and 20 berries for ‘Ochlockonee’ cultivars, based on availability.

### 4.4. Relative Gene Expression by Quantitative Real-Time RT-PCR (RT-qPCR)

RNA was extracted from three biological replicates of blueberry powder (0.4 g) following the protocol outlined by Yu et al. [67], with some modifications. cDNA synthesis was performed by reverse transcribing 0.7 μg of total RNA using the Maxima cDNA Kit with a dsDNase kit (Thermo Fisher Scientific, Waltham, MA, USA), adhering to the manufacturer’s guidelines. Transcript levels were measured via RT-qPCR on an iCycler iQ thermal cycler (Bio-Rad, Hercules, CA, USA), with quantification conducted using Real-Time Detection System Software (version 2.0). The amplification reactions were prepared in a total volume of 12 μL, consisting of 6 μL of NZY qPCR Green Master Mix (2×) (NZYTech Ltd., Lisbon, Portugal), 1 μL of each primer (10 μM), and 1 μL of cDNA. The PCR profile included an initial denaturation at 95 °C for 10 min, followed by 40 amplification cycles comprising 20 s denaturation at 95 °C and a 30 s annealing step at either 55 or 60 °C. Each biological replicate was analyzed with two technical replicates per gene. The Blueberry Genome Database (Genome Data Base for Vaccinium, GDV; vaccinium.org), which includes the *V. corymbosum* cv. Draper v1.0 genome, provided the gene sequences for expression analysis quantified using the 2^−ΔΔCT^ method, with *EF1* serving as the housekeeping gene. Gene-specific primers for RT-qPCR (Supplementary Table S1) were designed using Primer 3 software [68].

### 4.5. Statistical Analysis

All descriptive analyses were conducted using IBM-SPSS Statistics software, specifically version 28.0.0 (IBM Corp., Armonk, NY, USA). The data were analyzed using a one-way analysis of variance (ANOVA), followed by Tukey’s post-hoc test (*p* < 0.05). SPSS can conduct principal component analysis using both quantitative (scalar) and qualitative (categorical) data, including CATPCA for categorical variables. In this approach, qualitative attributes, mechanical parameters, and gene expression are considered unordered variables (multiple nominal), with each category represented by its corresponding number of states. All analyses were based on correlation matrices, and two dimensions were extracted to create scatterplots.

## 5. Conclusions

The efficacy of the gaseous treatments on the analyzed blueberries depended on the species. Thus, while the application of high levels of CO_2_ (15% and 20%) at low temperatures was effective in reducing decay in both cultivars, improvements in firmness were only observed in the highbush blueberry cv. ‘Duke’. In this cultivar, CO_2_ treatments modulated the expression of *XTH23*, *PL8*, and *GH5*, genes related to cell wall integrity. In contrast, the role of *PG*, and *PME* in maintaining firmness appeared minimal, with no significant differences between the two gaseous treatments. Although the application of high levels of CO_2_ did not affect the firmness of the rabbiteye blueberry cv. ‘Ochlockonee’, it effectively controlled fruit weight loss. Notably, a strong relationship was found between *XTH23* expression, the skin-breaking displacement parameter, and maturation index values, regardless of the postharvest response of the fruit. These differences in responses between species highlight the need to tailor postharvest treatments to specific blueberry species/cultivars. Overall, the results suggest that the application of short-term high CO_2_ treatments may be a viable strategy to prolong the postharvest life of highbush blueberries, thereby improving their quality.

## Figures and Tables

**Table 1:** Soluble solids content (SSC), titratable acidity (TA), SSC/TA ratio (MI), pH, and decay index of non-treated and 15% and 20% CO_2_-treated ‘Duke’ blueberries stored at 1.0 °C for 29 d.

		‘Duke’				
		SSC (°Brix)	TA (% Citric Acid)	MI (SSC/TA)	pH	Decay Index (%)
	0 d	9.17 ± 0.38 b	0.87 ± 0.01 b	10.58 ± 0.51 c	2.82 ± 0.01 a	0
Air	3 d	8.20 ± 0.17 a	1.03 ± 0.02 e	07.96 ± 0.26 a	2.96 ± 0.06 bc	0
	29 d	9.60 ± 0.00 b	0.66 ± 0.01 a	14.47 ± 0.13 d	3.24 ± 0.02 d	5.70 ± 0.04 c
15% CO_2_	3 d	8.37 ± 0.32 a	1.06 ± 0.02 f	07.92 ± 0.39 a	2.89 ± 0.04 ab	0
	29 d	8.30 ± 0.10 a	0.95 ± 0.01 d	08.71 ± 0.15 ab	2.95 ± 0.02 bc	4.43 ± 0.01 b
20% CO_2_	3 d	8.37 ± 0.25 a	0.92 ± 0.01 c	09.06 ± 0.32 b	3.01 ± 0.02 c	0
	29 d	8.43 ± 0.31 a	0.85 ± 0.00 b	09.92 ± 0.36 c	3.01 ± 0.01 c	2.35 ± 0.02 a

Values are the means of three replicate samples ± standard error. Different letters within each column indicate that means are statistically different according to the Tukey-b test (*p* < 0.05).

**Table 2:** Soluble solids content (SSC), titratable acidity (TA), SSC/TA ratio (MI), pH, and decay index of non-treated and 15% and 20% CO_2_-treated ‘Ochlockonee’ blueberries stored at 1.0 °C for 29 d.

		‘Ochlockonee’				
		SSC (°Brix)	TA (% Citric Acid)	MI (SSC/TA)	pH	Decay Index (%)
	0 d	12.67 ± 0.12 a	0.34 ± 0.02 b	37.70 ± 1.95 c	2.81 ± 0.01 b	0
Air	3 d	12.70 ± 0.00 a	0.45 ± 0.01 e	28.44 ± 0.37 a	2.85 ± 0.01 c	0
	29 d	13.87 ± 0.12 c	0.44 ± 0.01 de	31.53 ± 0.96 ab	2.87 ± 0.02 cd	2.03 ± 0.02 c
15% CO_2_	3 d	13.30 ± 0.10 b	0.32 ± 0.01 ab	41.15 ± 1.00 c	2.87 ± 0.01 cd	0
	29 d	13.33 ± 0.06 b	0.30 ± 0.02 a	45.03 ± 2.55 d	2.89 ± 0.01 d	1.62 ± 0.02 b
20% CO_2_	3 d	13.20 ± 0.00 b	0.39 ± 0.02 c	34.20 ± 1.80 b	2.76 ± 0.01 a	0
	29 d	12.70 ± 0.00 a	0.41 ± 0.01 cd	30.99 ± 0.76 ab	2.79 ± 0.03 ab	0.39 ± 0.01 a

Values are the means of three replicate samples ± standard error. Different letters within each column indicate that means are statistically different according to the Tukey-b test (*p* < 0.05).

**Table 3:** Mechanical properties of the penetration test corresponding to non-treated and 15% and 20% CO_2_-treated ‘Duke’ blueberries stored at 1.0 °C for 29 d.

		‘Duke’			
		Maximum Skin Breaking Force (N)	Slope at Skin Breaking (N/mm)	Displacement at Skin Breaking (mm)	Skin Breaking Work (mJ)
	0 d	1.95 ± 0.18 ab	0.60 ± 0.05 b–e	3.23 ± 0.28 ef	4.97 ± 0.59 b–d
Air	3 d	2.07 ± 0.16 a	0.68 ± 0.06 a	3.03 ± 0.28 f	5.39 ± 0.54 abc
	14 d	1.91 ± 0.16 a–d	0.63 ± 0.06 abc	3.02 ± 0.29 f	5.17 ± 0.49 a–d
	29 d	1.36 ± 0.13 f	0.29 ± 0.03 k	4.75 ± 0.79 a	4.25 ± 0.63 e
15% CO_2_	3 d	1.85 ± 0.17 b–e	0.61 ± 0.05 bcd	3.02 ± 0.25 f	5.21 ± 0.39 a–d
	14 d	1.69 ± 0.21 e	0.57 ± 0.05 c–g	2.92 ± 0.28 f	4.77 ± 0.47 cde
	29 d	1.69 ± 0.16 e	0.41 ± 0.04 ij	4.09 ± 0.40 bc	5.08 ± 0.48 bcd
20% CO_2_	3 d	1.68 ± 0.13 e	0.54 ± 0.05 d–g	3.04 ± 0.27 f	4.61 ± 0.45 de
	14 d	1.77 ± 0.16 b–e	0.54 ± 0.05 efg	3.22 ± 0.28 ef	4.88 ± 0.46 b–e
	29 d	1.71 ± 0.18 de	0.40 ± 0.04 j	4.26 ± 0.44 b	5.08 ± 0.52 bcd

Values are the means of fifteen replicate samples ± standard error. Different letters within each column indicate that means are statistically different according to the Tukey-b test (*p* < 0.05).

**Table 4:** Mechanical properties of the penetration test corresponding to non-treated and 15% and 20% CO_2_-treated ‘Ochlockonee’ blueberries stored at 1.0 °C for 29 d.

		‘Ochlockonee’			
		Maximum Skin Breaking Force (N)	Slope at Skin Breaking (N/mm)	Displacement at Skin Breaking (mm)	Skin Breaking Work (mJ)
	0 d	1.43 ± 0.12 ab	0.49 ± 0.04 bc	2.91 ± 0.26 fgh	3.92 ± 0.40 abc
Air	3 d	1.51 ± 0.15 a	0.56 ± 0.05 a	2.68 ± 0.28 gh	4.27 ± 0.41 a
	14 d	1.45 ± 0.17 ab	0.41 ± 0.03 def	3.51 ± 0.50 cde	3.91 ± 0.52 abc
	29 d	1.41 ± 0.19 ab	0.38 ± 0.06 d–g	3.78 ± 0.59 abc	3.75 ± 0.61 abc
15% CO_2_	3 d	1.35 ± 0.13 ab	0.54 ± 0.05 ab	2.49 ± 0.24 h	3.76 ± 0.40 abc
	14 d	1.45 ± 0.19 ab	0.41 ± 0.06 def	3.51 ± 0.45 cde	3.76 ± 0.59 abc
	29 d	1.34 ± 0.23 ab	0.33 ± 0.07 g	4.15 ± 0.74 a	3.59 ± 0.71 c
20% CO_2_	3 d	1.50 ± 0.15 a	0.55 ± 0.05 a	2.69 ± 0.29 gh	4.23 ± 0.39 ab
	14 d	1.39 ± 0.17 ab	0.39 ± 0.05 d–g	3.56 ± 0.38 b–e	3.79 ± 0.59 abc
	29 d	1.35 ± 0.20 ab	0.39 ± 0.08 d–g	3.53 ± 0.53 b–e	3.61 ± 0.61 c

Values are the means of twenty replicate samples ± standard error. Different letters within each column indicate that means are statistically different according to the Tukey-b test (*p* < 0.05).

## Data Availability

Data are contained within the article or Supplementary Materials.

## Supplementary Materials

The following supporting information can be downloaded at: https://www.mdpi.com/article/10.3390/plants13233398/s1, Figure S1: Biplot-CATPCA using 'Duke' blueberry results of gene expression (XTH23, PL8, PG, PME3, EXP4, GH5), mechanical properties (MSBreaking, SlopeSB, DisplacementSB, SBWork), total decay, water loss (WLoss) and maturation index (MI) as variables. The component loadings of the variables are indicated by black lines, while the analyzed samples are represented by blue dots: [(1) freshly harvested; (2) 3 d Air, (3) 3 d 15% CO_2_, (4) 3 d 20% CO_2_, (5) 14 d Air, (6) 3 d 15% CO_2_ + 11 d Air, (7) 3 d 20% CO_2_ + 11 d Air, (8) 29 d Air, (9) 3 d 15% CO_2_ + 26 d Air, (10) 3 d 20% CO_2_ + 26 d Air]; Figure S2: Biplot-CATPCA using 'Ochlockonee' blueberry results of gene expression (XTH23, PL8, PG, PME3, EXP4, GH5), mechanical properties (MSBreaking, SlopeSB, DisplacementSB, SBWork), total decay, water loss (WLoss) and maturation index (MI) as variables. The component loadings of the variables are represented by black lines, and the analyzed samples are depicted by blue dots: [(1) freshly harvested; (2) 3 d Air, (3) 3 d 15% CO_2_, (4) 3 d 20% CO_2_, (5) 14 d Air, (6) 3 d 15% CO_2_ + 11 d Air, (7) 3 d 20% CO_2_ + 11 d Air, (8) 29 d Air, (9) 3 d 15% CO_2_ + 26 d Air, (10) 3 d 20% CO_2_ + 26 d Air]; Table S1: Primers used for the quantification of gene expression levels by RT-qPCR.
