# Supplementary material for: Effect of Short-Term High-CO2 Treatments on the Quality of Highbush and Rabbiteye Blueberries During Cold Storage
Source: Plants (Basel). 2024 Dec 3;13(23):3398. doi: 10.3390/plants13233398 (PMC11644509; doi:10.3390/plants13233398)
Supplement: Supplementary file 1 [file plants-13-03398-s001.zip › Supplementary Figure S2.pptx]

## Slide 1
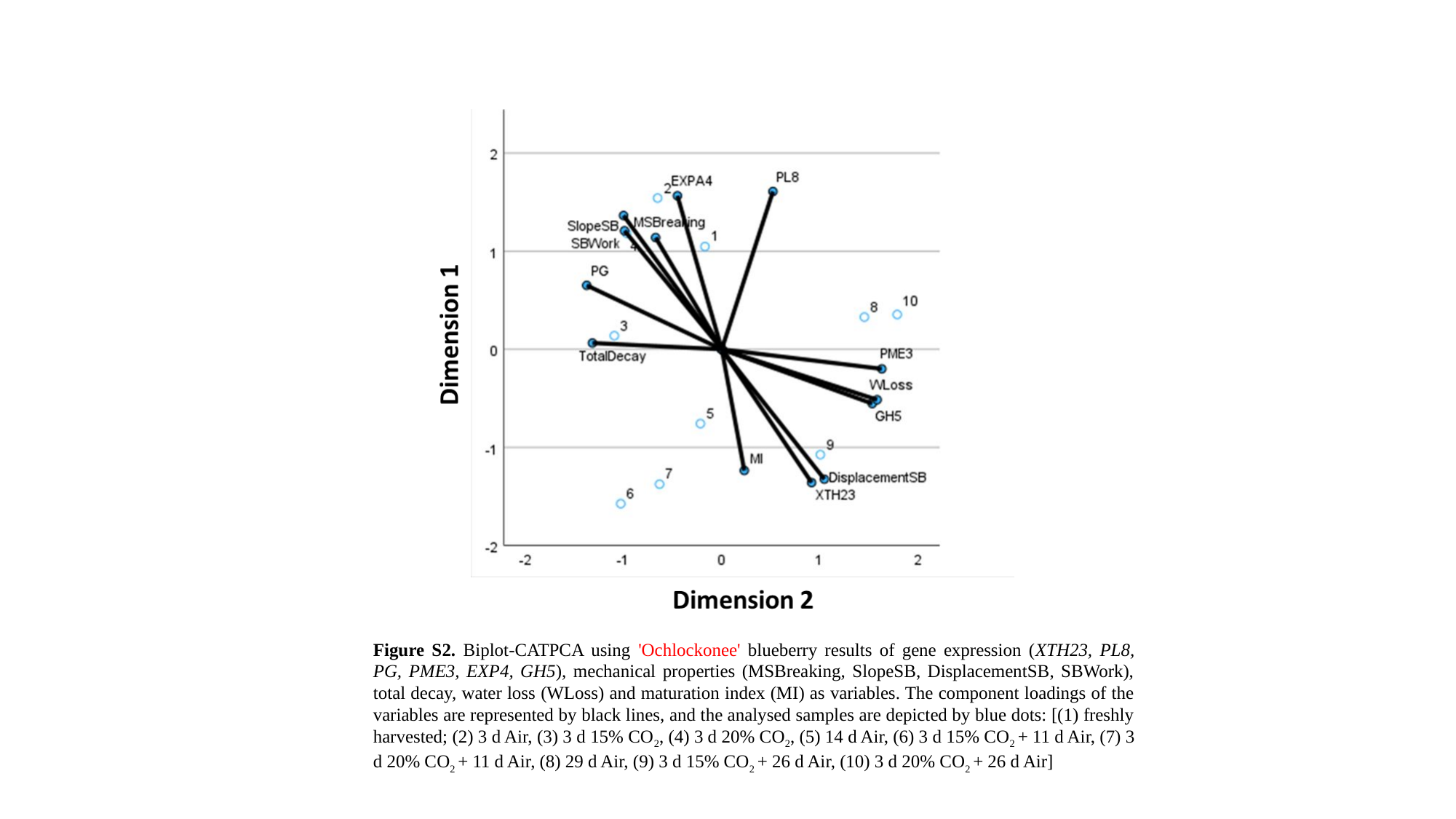

Figure S2. Biplot-CATPCA using 'Ochlockonee' blueberry results of gene expression (XTH23, PL8, PG, PME3, EXP4, GH5), mechanical properties (MSBreaking, SlopeSB, DisplacementSB, SBWork), total decay, water loss (WLoss) and maturation index (MI) as variables. The component loadings of the variables are represented by black lines, and the analysed samples are depicted by blue dots: [(1) freshly harvested; (2) 3 d Air, (3) 3 d 15% CO2, (4) 3 d 20% CO2, (5) 14 d Air, (6) 3 d 15% CO2 + 11 d Air, (7) 3 d 20% CO2 + 11 d Air, (8) 29 d Air, (9) 3 d 15% CO2 + 26 d Air, (10) 3 d 20% CO2 + 26 d Air]
